# Genome-Wide Identification of the Vacuolar H^+^-ATPase Gene Family in Five Rosaceae Species and Expression Analysis in Pear (*Pyrus bretschneideri*)

**DOI:** 10.3390/plants9121661

**Published:** 2020-11-27

**Authors:** Hongsheng Zhou, Wen Huang, Shufen Luo, Huali Hu, Yingtong Zhang, Leigang Zhang, Pengxia Li

**Affiliations:** 1 Institute of Agricultural Facilities and Equipment, Jiangsu Academy of Agricultural Sciences, Nanjing 210014, China; hongshengzhou@jaas.ac.cn (H.Z.); 20140013@jaas.ac.cn (S.L.); 20070023@jaas.ac.cn (H.H.); 20180002@jaas.ac.cn (Y.Z.); 2 Jiangsu Key Laboratory for Horticultural Crop Genetic Improvement, Nanjing 210014, China; 3 Nanjing Institute of Vegetable Science, Nanjing 210042, China; xiaohuangwen588@163.com; 4 School of Food and Biological Engineering, Jiangsu University, Zhenjiang 212013, China

**Keywords:** bioinformatics analysis, development, expression profile, Rosaceae species, V-ATPase

## Abstract

Vacuolar H^+^-ATPases (V-ATPase) are multi-subunit complexes that function as ATP hydrolysis-driven proton pumps. They play pivotal roles in physiological processes, such as development, metabolism, stress, and growth. However, there have been very few studies on the characterisation of V-ATPase (VHA) genes in Rosaceae species. Therefore, in the present study, we performed a genome-wide analysis and identified *VHA* gene family members in five Rosaceae species (*Pyrus bretschneideri*, *Malus domestica*, *Prunus persica*, *Fragaria vesca,* and *Prunus mume*). A total of 159 *VHA* genes were identified, and were classified into 13 subfamilies according to the phylogenetic analysis. The structure of VHA proteins revealed high similarity among different *VHA* genes within the same subgroup. Gene duplication event analysis revealed that whole-genome duplications represented the major pathway for expansion of the *Pyrus bretschneideri VHA* genes (*PbrVHA* genes). The tissue-specific expression analysis of the pear showed that 36 *PbrVHA* genes were expressed in major tissues. Seven *PbrVHA* genes were significantly downregulated when the pollen tube growth stopped. Moreover, many *PbrVHA* genes were differentially expressed during fruit development and storage, suggesting that *VHA* genes play specific roles in development and senescence. The present study provides fundamental information for further elucidating the potential roles of *VHA* genes during development and senescence.

## 1. Introduction

Vacuoles are the largest compartments within plant cells, occupying up to 90% of the cellular volume [1]. In plants, vacuoles play important roles in growth and development and serve various functions, including storage and transport, cell homoeostasis, and stress response [2]. All vacuolar functions require enormous fluxes of metabolites and ions, which are transported by a series of vacuolar transport proteins [3]. Transport across the tonoplast is primarily energized by the ATP hydrolysis-driven proton pumps vacuolar H^+^-ATPases (V-ATPases) [3,4,5]. V-ATPases are highly conserved protein complexes, which are present in the vacuole as well as other intracellular compartments [5]. V-ATPases are large multi-subunit complexes comprising the cytosolic V_1_ domain and the membrane-integral V_0_ domain [6,7]. The two domains are joined by a stalk domain, which makes them a complex with integrated structure and function. The V_1_ domain harbors eight subunits (VHA-A, B, C, D, E, F, G, and H) and is responsible for ATP hydrolysis. The V_0_ domain consists of up to five subunits (VHA-a, c, c”, d, and e) and is involved in proton transport [6]. V-ATPases are the key regulators of functions of the vacuole and other endomembrane compartments in plants [8,9].

Research on plant V-ATPases began nearly 40 years ago, when the enzyme was discovered [10,11]. V-ATPase is a proton pump present in the vacuole and other intracellular compartments, including the endoplasmic reticulum (ER), Golgi, vesicles, and endosomes [6,12]. Moreover, V-ATPase activity in the trans-Golgi network/early endosome is essential for functional secretion and recycling [13], whereas V-ATPase activity in the tonoplast is required for ion homoeostasis and nutrient transport [3,14]. *VHA* genes, such as *AtVHA-c5*, *AtVHA-d2*, and *JrVHAG1*, have been speculated to be involved in stress response [11,15,16]. Likewise, other members of this gene family, such as *PutVHA-c*, *AtVHA-a2*, *AtVHA-a3*, and *AtVHA-B2*, are associated with plant growth or senescence [14,17]. To summarize, V-ATPases are involved in various physiological processes, including cell expansion, membrane trafficking, nutrient accumulation, leaf senescence, and environmental stress tolerance [17,18,19].

In the past decades, genes encoding various *VHA* subunits in plants have been identified, including 28 *VHA* genes in *Arabidopsis thaliana* [6], 54 *VHA* genes in *Glycine max* [12], 23 *VHA* genes in tomato [20], and 51 *VHA* genes in citrus fruit [18]. Moreover, *VHA* genes are conserved among green plants, although their number varies across different species [9,12]. V-ATPase has a highly complex subunit composition, and genes encoding at least 13 distinct subunits have been identified thus far [12]. Unlike P-type proton pumps encoded by multiple genes [21], most V_1_ subunits (A, C, D, F, and H) of V-ATPase are encoded by a single gene, whereas V_0_ subunits are encoded by multiple genes in *Arabidopsis* and some higher plant species [6,12]. Recently, genome databases have been used to identify *VHA* genes in numerous plant species, and genes encoding the V-ATPase subunit H in 11 major crops have been identified [22]. However, genome-wide identification and bioinformatic analysis of *VHA* genes have not been performed in Rosaceae species.

The Rosaceae family is an important family with an extraordinary spectrum of fruit types, including pear (*Pyrus bretschneideri*), apple (*Malus x domestica*), peach (*Prunus persica*), plum (*Prunus mume*), strawberry (*Fragaria vesca*) and so on [23]. Remarkably, a few biological functions of V-ATPases have been discovered in several Rosaceae species. Proteomic analysis revealed that abundance of the V-ATPase protein decreased during fruit senescence in apple and strawberry [24,25]. Moreover, the activity of V-ATPases and transcript expression patterns of the corresponding genes were upregulated during storage following 1-methylcyclopropene treatment of apple fruit [26]. Recently, the genome sequences of five main Rosaceae species (*Pyrus bretschneideri*, *Malus x domestica*, *Prunus persica*, *Prunus mume* and *Fragaria vesca*) had been published [27,28,29]. However, limited information is available regarding the roles of *VHA* genes in Rosaceae species. Therefore, identification and analyses of *VHA* genes in Rosaceae species are of great significance. Pear is one of the most important commercial fruits in the Rosaceae family and multiple transcriptome data could be gathered for this species. Pear is an excellent model for studying fruit development and senescence. In the present study, the V-ATPase (*VHA*) family genes in five Rosaceae species were identified, and the gene structure, conserved motifs, chromosomal distribution, evolution, gene ontology and expression patterns were simultaneously analysed in pear. The results of this study will help elucidate the biological functions and molecular mechanisms of action of the *VHA* gene family in several Rosaceae species.

## 2. Results

### 2.1. Identification of V-ATPase (VHA) Genes in Five Rosaceae Species

In *Arabidopsis*, 28 *VHA* genes encoding different V-ATPase subunits have been identified [6]. To identify and comprehensively analyze *VHA* genes in five Rosaceae species, sequences of *A. thaliana* were used to query the corresponding genome. Pfam and SMART databases were used to search for the conserved domain of each V-ATPase subunit. After filtering for domain integrity and redundancy of the candidate genes, 159 *VHA* genes were identified, including 48 in apple (*Malus x domestica*), 43 in Chinese white pear (*Pyrus bretschneideri*), 25 in Chinese plum ( *Prunus mume*), 23 in peach (*Prunus persica*), and 20 in wild strawberry (*Fragaria vesca*) (Table 1). Based on their similarity to the *Arabidopsis VHA* genes, the identified genes were preliminarily named *VHA-x*, where x represents the letter code for each subunit. Evidently, the Rosaceae genome database comprised at least one gene encoding one V-ATPase subunit. Detailed information on *VHA* genes, including name, identifier (ID), chromosomal distribution, exon number, and length, is provided in Table S1.

### 2.2. Phylogenetic Analysis and haracterisation of VHA Genes

**Figure 1:**
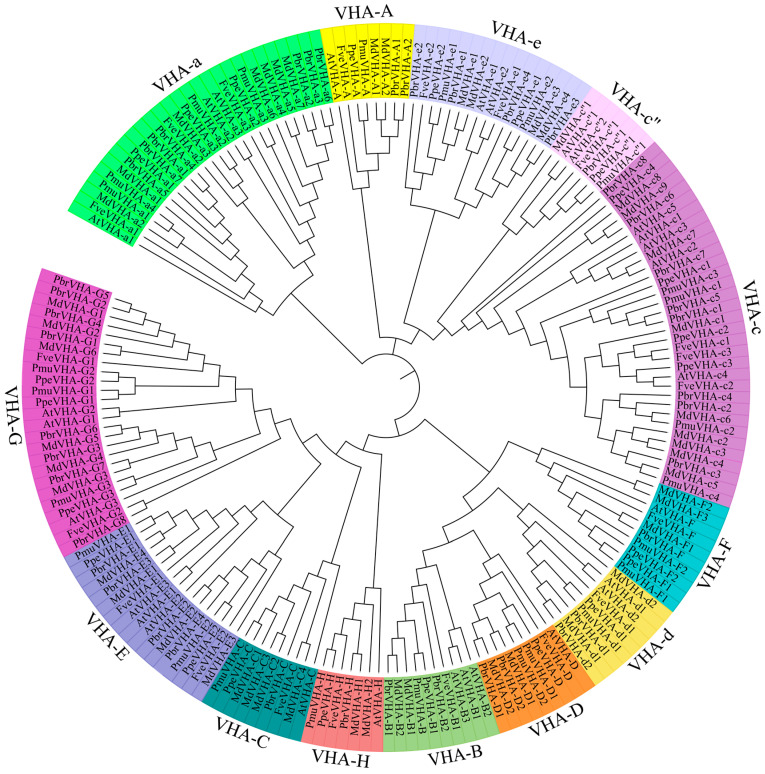
Phylogenetic analysis of V-ATPase proteins from *Arabidopsis* and five Rosaceae species (*Fragaria vesca, Prunus persica, Prunus mume, Pyrus bretschneideri* and *Malus x domestica*). The phylogenetic tree was constructed using MEGA6.0 software (http://www.megasoftware.net/) by neighbor-joining method and diagram was drawn using online tool (iTOL, https://itol.embl.de/). The different colour indicate different subfamily respectively.

To further classify the VHA proteins, a neighbor-joining phylogenetic tree of VHA-like proteins in *Arabidopsis* and the five Rosaceae species was constructed. Phylogenetic analysis of the 159 genes in Rosaceae species, along with 28 genes in *Arabidopsis*, was performed across 13 subfamilies, namely VHA-A, VHA-B, VHA-C, VHA-D, VHA-E, VHA-F, VHA-G, VHA-H, VHA-a, VHA-c, VHA-c”, VHA-d, and VHA-e (Figure 1). The number of VHA protein family were divided in 13 well-conserved groups. The clades A, B, C, D, E, F, G, H, a, c, c”, d, and e contained 7, 7, 8, 8, 14, 9, 22, 6, 24, 28, 5, 7, and 14 members, respectively (Figure 1). VHA-c” contained the lowest number of *VHA* genes (*n* = 5), while VHA-c contained the highest number of *VHA* genes (*n* = 28). All VHA proteins belong to two V-ATPase subunits (V_0_ and V_1_) (Table 1), indicating the diverse functions of *VHA* gene in Rosaceae species. Detailed characteristics of *VHA* genes are provided in Table S1.

Pear is a typical Rosaceae species, which represents an excellent model for studying fruit development. The number of *VHA* genes in pear and apple were almost double the number in other analyzed species (Figure 1), suggested that it is necessary to make a systematic study in the complexity of *PbrVHA* genes. Furthermore, multiple transcriptome data are available for pear in the NCBI databases. Therefore, the *VHA* genes in five Rosaceae species were identified, and the gene structure, conserved motifs, chromosomal distribution, evolution, and expression patterns were emphatically analyzed in pear. Average length of the PbrVHA proteins was 70 (PbrVHA-e) to 868 (PbrVHA-a5) amino acids. Molecular weight of the PbrVHA proteins was 7584.44 (PbrVHA-e1) to 99,317 (PbrVHA-a5) Da, and their isoelectric points (pI) were 4.9 (PbrVHA-d2) to 9.61 (PbrVHA-c5) (Table S1).

### 2.3. Gene Structure, Conserved Motif, and Phylogenetic Analysis of the PbrVHA Genes

**Figure 2:**
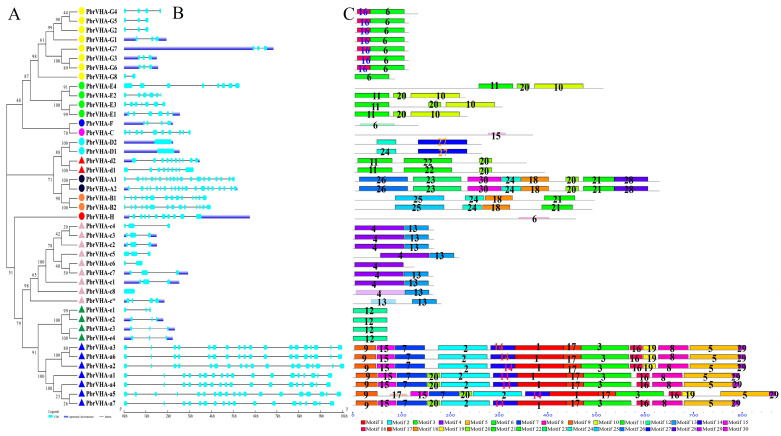
Structural and phylogenetic analyses of *PbrVHA* genes. (**A**) A phylogenetic tree based on sequences of the PbrVHA proteins was constructed using MEGA 6.0 (**B**) The intron–exon structures were depicted by Gene Structure Display Server 2.0. UTRs and exons are represented by blue and light blue boxes, respectively, and introns are represented by grey lines. (**C**) Distribution of conserved motifs of PbrVHA proteins was described by Motif-based sequence analysis tools. Different motifs are indicated by different colours and numbered from 1 to 30; black lines represent non-conserved sequences.

An unrooted tree was constructed to further explore the phylogeny of *PbrVHA* genes (Figure 2A). Consistent with the phylogenetic tree of *Arabidopsis*, our analysis supported the classification of *PbrVHA* genes into 13 subfamilies. Moreover, the exon–intron structure was analyzed based on genomic and cDNA sequences of *PbrVHA* genes. The structure of *PbrVHA* genes was relatively conserved within the same subfamily but varied across different subfamilies (Figure 2B). For instance, exon number in *VHA* genes ranged from 1 to 20, although the same subfamily generally had the same or similar exon number. Next, structures of conserved motifs of the 43 PbrVHA proteins were predicted using Multiple Em for Motif Elicitation (MEME) (Figure 2C). The block diagram and logos of 30 conserved motifs are shown in Figure S1. Motifs of the VHA proteins were mostly conserved within the same subfamily but varied across different subfamilies (Figure 2C). Motifs 1, 2, 3, 5, 7, 9, 14, 15, 16, 17, and 29 were found in all PbrVHA-a proteins, while motif 12 was present in the PbrVHA-G protein alone.

The spatial structure of proteins plays a role in the biological function of proteins. Further, the secondary structures of these proteins were predicted using the online tool NPS@SOPMA (Figure S2A). The results showed that most VHA proteins were predicted to contain alpha helix, except for PbrVHA-G2, PbrVHA-G5, and PbrVHA-G8. Previous investigations suggested that the ATP-binding region on the A subunit may play an important role, so that one P-loop (GXXXXGKT/S) was identified in Figure S2B. It was predicted that proteins of VHA-a, VHA-c, VHA-c”, and VHA-e groups contained 2–8 cross-membrane domains (Figure S2). We also found that there were conserved proton-binding sites located in the fourth transmembrane domain of VHA-c and VHA-c” protein (Figure S2C,D). Similar to the motif analysis, the 3D structure analysis of the VHA proteins also showed that they shared high 3D structure similarity within the same subfamily (Figure S3). Thus, these results provided important information of their potential cellular roles.

### 2.4. Chromosomal Distribution and Gene Duplication of PbrVHA Genes

**Figure 3:**
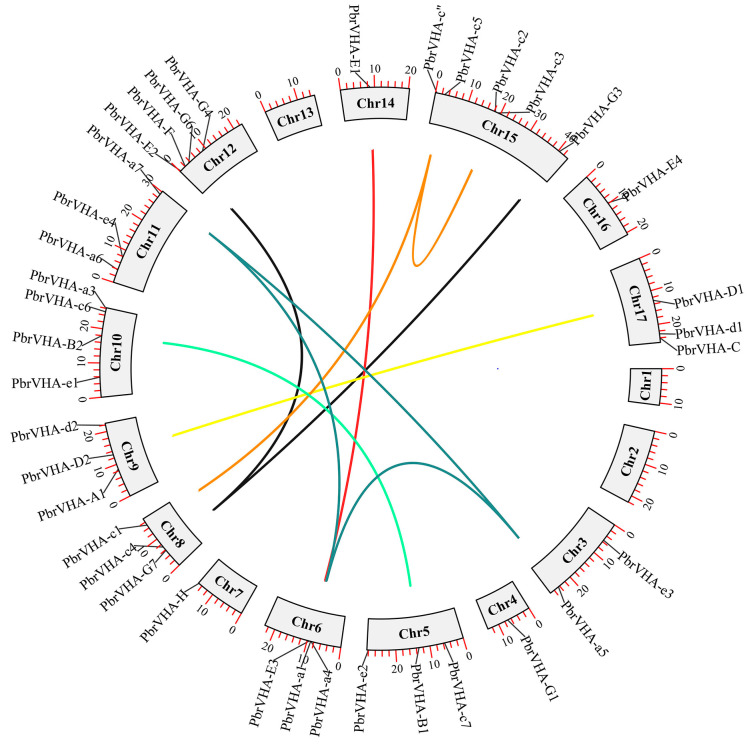
Chromosomal distribution and synteny analysis of the *PbrVHA* genes. Gene pairs with a syntenic relationship were joined by a line. Chromosome number was indicated on the inner side.

Chromosomal distribution and synteny of *PbrVHA* genes were analyzed to better understand the locations and duplication events in *PbrVHA* genes. A total of 38 *PbrVHA* genes were distributed on 14 of the 17 chromosomes (Figure 3), and 6 genes were located on scaffolds. The distribution of *PbrVHA* genes was random and uneven, and most *PbrVHA* genes were found on chromosomes 15 (*n* = 5), 10 (*n* = 4), and 12 (*n* = 4). Similarly, *VHA* genes in the other four species were randomly distributed (Figure S4). A total of 26 syntenic gene pairs were identified among the five Rosaceae species, including 10 in pear (Figure 3), 8 in apple (Figure S4A), 4 in peach (Figure S4B), 3 in Chinese plum (Figure S4C), and 1 in strawberry (Figure S4D). To further clarify their evolutionary history, we analyzed the orthologous *VHA* genes among five Rosaceae species. A total of 109 orthologous *VHA* gene pairs were identified, including 24, 37, 19, and 29 collinear gene pairs between pear and strawberry, apple, Chinese plum, and peach, respectively (Figure S5).

To clarify the driving forces of gene duplication, Ka, Ks, and Ka/Ks values were calculated for paralogous gene pairs in pear (Table 2). The Ka/Ks value indicates selection pressure on duplicated *PbrVHA* genes during the course of evolution. Ka/Ks values of all syntenic *PbrVHA* gene pairs were less than 0.3, indicating that these genes are under the influence of purifying selection. Given their importance in the expansion of gene families, duplication events in paralogous gene pairs were analyzed. All 10 gene pairs were whole-genome duplication (WGD)-derived, suggesting that WGD represents the principal pathways for gene expansion of the *PbrVHA* gene family.

### 2.5. Gene Ontology (GO) Analysis of PbrVHA Proteins

**Figure 4:**
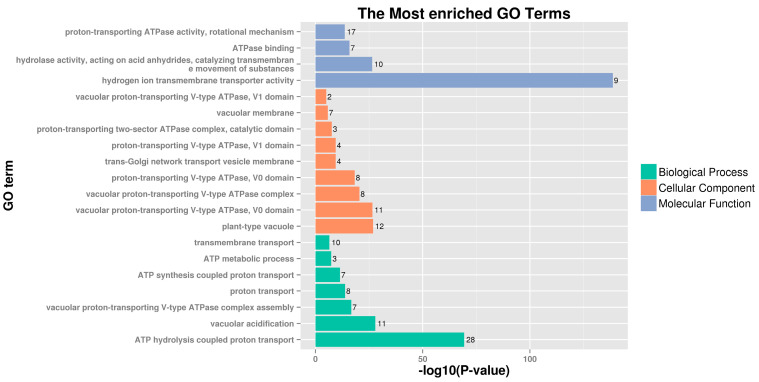
Gene ontology analysis of PbrVHA proteins in biological processes, molecular functions, and cellular component categories.

To further understand the functions of the PbrVHA proteins, GO annotation and GO enrichment analyses were performed. Our results showed that PbrVHA proteins were most enriched for 20 GO terms (Figure 4), which were divided into three categories: biological process (BP), cellular component (CC), and molecular function (MF). GO terms related to ATP hydrolysis coupled proton transport (GO:0015991), proton-transporting ATPase activity (GO:0046961), plant-type vacuole (GO:0000325), vacuolar proton-transporting V-type ATPase, V_0_ domain (GO:0000220), hydrogen ion transmembrane transporter activity (GO:0015078), and vacuolar acidification (GO:0007035), were obviously enriched GO categories from three categories. The GO enrichment suggested that *PbrVHA* genes play important roles in vacuole and trans-Golgi network. Furthermore, these results provide useful information for future gene characterization studies in pear.

### 2.6. Gene Expression Patterns of PbrVHA Genes in Different Tissues and During Fruit Development

**Figure 5:**
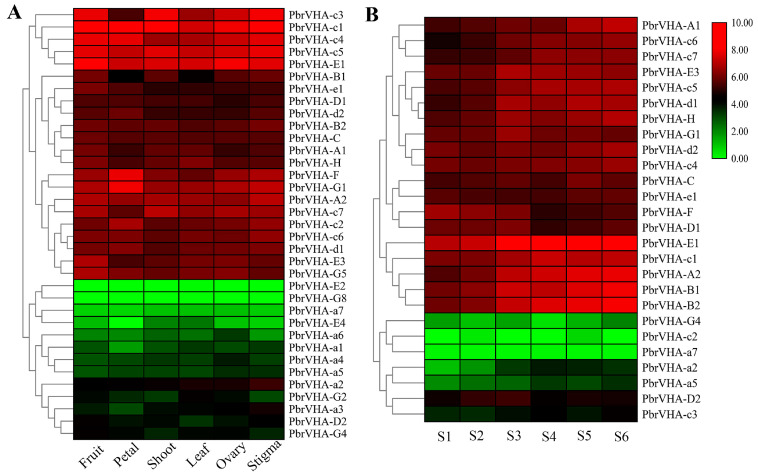
Expression patterns of *PbrVHA* genes in different tissues and during fruit development of “Yali” pear. (**A**) Expression patterns of *PbrVHA* genes in six different tissues, including fruit, petal, shoot, leaf, ovary, and stigma. (**B**) Expression profiles of *PbrVHA* genes during fruit development, including the fruit-setting stage (S1), physiological fruit dropping stage (S2), fruit rapid enlargement stage (S3), a month after fruit enlargement stage (S4), pre-mature stage (S5), and mature stage (S6). The expression levels were calculated as log2-transformed (RPKM + 1) values. Green, black, and red indicate low, moderate, and high levels, respectively.

To explore the putative functions of *PbrVHA* genes, their expression patterns in six major tissues (fruit, petal, shoot, leaf, ovary, and stigma) were analyzed based on previously reported RNA-Seq data [30]. Seven genes were not expressed or were weakly expressed in all tissues, which is probably explained by the presence of uncharacterized pseudogenes or specific gene expression at a particular stage. Moreover, 36 *PbrVHA* genes were expressed in major tissues and clustered into six subsets based on their expression patterns (Figure 5A). Among these genes, *PbrVHA-c3*, *PbrVHA-c1*, *PbrVHA-c4*, *PbrVHA-c5*, and *PbrVHA-E1* exhibited the highest transcript abundances in different tissues, whereas *PbrVHA-E2*, *PbrVHA-G8*, and *PbrVHA-a7* exhibited lower transcript abundances in six tissues (Figure 5A). Several genes were highly expressed in specific tissues, such as *PbrVHA-F* and *PbrVHA-G1* in petal tissues. To further explore the possible roles of *PbrVHA* genes in fruit development, expression patterns of 26 genes at six different fruit developmental stages were evaluated based on publicly available RNA-Seq data [31]. The expression of *PbrVHA-B2* showed an increasing trend throughout fruit maturity, while the expression of *PbrVHA-G1*, *PbrVHA-E3*, *PbrVHA-E1*, and *PbrVHA-d1* was upregulated at the S3 stage (Figure 5B). Interestingly, *PbrVHA-D1*, *PbrVHA-D2*, *PbrVHA-E1*, *PbrVHA-F*, and *PbrVHA-G1* showed relatively high expression levels at the S6 stage (Figure 5B), indicating their crucial role in fruit ripening. Moreover, the expression profiles of *PbrVHA*s during fruit development were cultivar dependent (Figure S6).

### 2.7. Expression Profiles of PbrVHA Genes during Pollen Tube Development and Fruit Senescence

**Figure 6:**
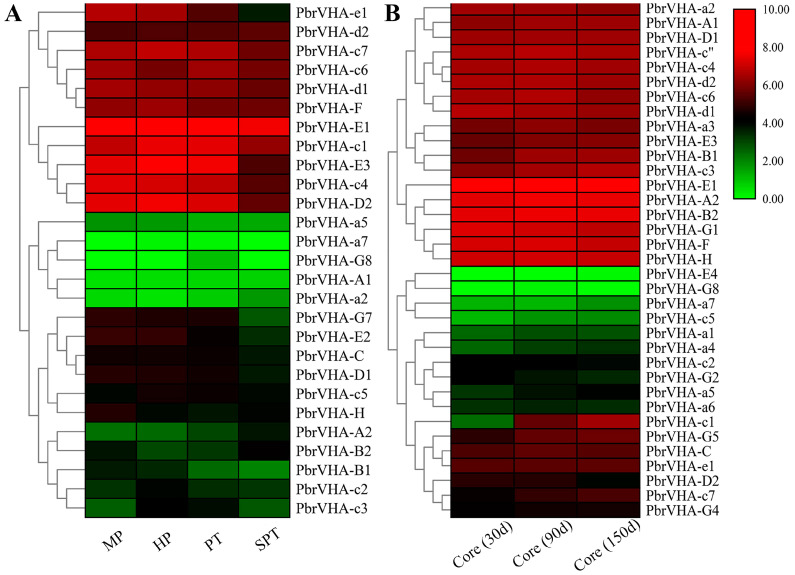
Expression profiles of *PbrVHA* genes during pollen tube development and fruit senescence. (**A**) The expression of *PbrVHA* genes during four development stages of pear pollen, including mature pollen grains (MP), hydrated pollen grains (HP), growing pollen tubes (PT) and stopped-growth pollen tubes (SPT). (**B**) Expression profiles of *PbrVHA* genes in the pear core during fruit storage at three stage (30d, 90d and 150d after harvesting). Color scale represents log2-transformed FPKM + 1 values. Green, black, and red indicate low, moderate, and high levels, respectively.

To explore the possible functions of *PbrVHA* genes, their expression patterns during pollen tube development [32] and fruit senescence [31] were also analyzed. A total of 27 *PbrVHA* genes were expressed during pollen tube growth and senescence, with *PbrVHA-G8* specifically expressed in the growing pollen tube (Figure 6A). Moreover, seven *PbrVHA* genes (*PbrVHA-c1*, *PbrVHA-c3*, *PbrVHA-c4*, *PbrVHA-D2*, *PbrVHA-E3*, *PbrVHA-e1*, and *PbrVHA-G7*) were significantly downregulated when the pollen tube growth stopped, suggesting their important roles in pollen tube growth. *PbrVHA-c3* was highly expressed during the hydrous and growth stages (Figure 6A), suggesting that it positively regulates pollen tube growth. To analyze whether *PbrVHA* is involved in core browning, we compared the transcript levels of *PbrVHA* genes during browning in “Whangkeumbae” pear [33]. Two *PbrVHA*s (*PbrVHA-c1* and *PbrVHA-D2*) were differentially expressed during browning (Figure 6B), suggesting that these genes function in core browning. To further explore the potential roles of *PbrVHA* genes during fruit senescence, their expression profiles during postharvest storage were analyzed [31]. A total of 21 *PbrVHA* genes were transcribed during the storage of “Housui” pear, with diverse expression patterns (Figure S7). The expression levels of 10 genes (*PbrVHA-G4*, *PbrVHA-G1*, *PbrVHA-E3*, *PbrVHA-E1*, *PbrVHA-F*, *PbrVHA-C*, *PbrVHA-D2*, *PbrVHA-A1*, *PbrVHA-H*, and *PbrVHA-a7*) were significantly reduced in “Housui” pear (Figure S7). Moreover, the expression profiles of *PbrVHA* genes during fruit senescence were cultivar dependent (Figure S7).

## 3. Discussion

V-ATPases are found in a wide variety of endomembrane system of all eukaryotes, which function as ATP hydrolysis-driven proton pumps [5]. With extensive research, an increasing number of biological functions of *VHA* genes in plants are gradually being discovered [14,19]. V-ATPases energize secondary active transport and serve as important regulators of membrane trafficking [5]. Moreover, V-ATPases play vital roles in plant development and stress response, functioning as a house-keeping and stress-responsive enzyme [22]. Whole-genome sequencing has enabled the investigation of *VHA* genes and their potential functions. The *VHA* gene families in *A. thaliana*, rice, and tomato, among other plants, have been described [6,12,20]. However, there has been no comprehensive study on genome-wide identification of *VHA* genes in Rosaceae species.

The present study is the first to reveal detailed characteristics of the *VHA* gene family in Rosaceae species. A total of 159 *VHA* genes were identified in apple (*n* = 48), pear (*n* = 43), Chinese plum (*n* = 25), peach (*n* = 23), and strawberry (*n* = 20). Our results indicate that the number of genes encoding V-ATPase proteins may vary across plant species. The similar number of VHA proteins between pear and apple may indicate the parallel evolutionary relationship of this gene family between these two species [27,34]. Phylogenetic analysis showed that the VHA proteins of the Rosaceae species were clustered with the 13 previously described, corresponding subfamilies in *Arabidopsis* [6], confirming the suitability of our methods of gene identification and phylogenetic tree construction.

To explore the functions of *PbrVHA* genes, phylogeny, exon–intron structures, conserved motif structures, and spatial structure were analyzed in this research. PbrVHA proteins were classified into 13 subfamilies, consistent with the previously reported classification of *A. thaliana* [12]. The number and distribution of introns and exons were mostly conserved within the same subfamily but varied across different subfamily (Figure 2B). MEME analysis showed that VHA proteins within the same subfamily contained the same basal motifs (Figure 2C). Therefore, VHA proteins in the same subfamily may show similar structures and functions. Accordingly, the functions of *VHA* genes in Rosaceae species can be predicted based on the known functions of homologous genes in other species. For example, overexpression of *VHA-B* from the halophyte *Halostachys caspica* enhanced salt tolerance of transgenic *Arabidopsis* [35], indicating that its homologous gene may serve similar functions. Apple V-ATPase subunit B1 is involved in drought and salt tolerance [36], which further supports our prediction. The localization of the V-ATPase protein was previously shown to be determined by the isoforms of subunit a (VHA-a) in *Arabidopsis* [3,11]. It was predicted that PbrVHA-a subunit that consists of a C-terminal hydrophobic domain with six transmembrane domains (Figure S2), suggested that PbrVHA-a subunit may be the determinant that targets the V-ATPase to the TGN/EE or to the tonoplast as previously described [11]. The assembly of V-ATPases requires the presence of all V_1_ and V_0_ subunits [7], so that it is very important to identify the functional domain of every subunit. Conserved proton-binding sites were identified in the fourth transmembrane domain of VHA-c and VHA-c” protein, suggested that subunit c and its isoforms c” may form a H^+^-binding rotor ring in H^+^-transporting V-ATPases [7,8]. The V_0_ subunits are integral membrane proteins except for subunit d, which is consistent with the number of transmembrane domains in this research.

Gene duplication is an important evolutionary event for the expansion of gene families. Gene duplication can be classified into five types, including WGD, tandem duplication, proximal duplication, dispersed duplication, and transposed duplication [37]. Our results indicated that *VHA* genes have expanded mainly through WGD, which contributes to expansion of *PbrVHA* genes (Table 2). WGD may greatly contribute to the evolution of physiological diversity in pear [37]. The number of *VHA* genes in pears and apples was much higher than that in peach, strawberry, and Chinese plum (Table 1). These findings corroborate the previous report that pear and apple may have undergone a recent lineage-specific WGD, while strawberry does not share a recent WGD event [27]. In previous studies, the Ka/Ks values of various gene pairs were variable, while WGD genes are usually conserved with smaller Ka/Ks ratios [37]. Ka/Ks values of all paralogous *VHA* gene pairs were less than 0.3 (Table 2), suggesting that these gene pairs have undergone strong purifying selection pressure [38]. Together, these results indicate that PbrVHA proteins are highly conserved and that purifying selection plays a key role in the expansion of the *PbrVHA* gene family.

The roles of *VHA* genes have been studied previously. These genes are involved in numerous biological processes, including development, metabolism, and stress response [14,35]. VHA-c5 (*Arabidopsis*), VHA-C (*Arabidopsis*), VHA-E (Wheat), VHA-B1 (Apple), and VHA-G1 (*Juglans regia*) are involved in plant responses to abiotic stress [15,39,40]. Based on GO analysis, *PbrVHA* genes were involved in proton transport, vacuolar acidification, transmembrane transport, and so on, which is consistent with previous research about the function of *VHA* genes [12,41]. Gene expression patterns are usually consistent with gene functions. In this study, transcriptomic analysis was conducted to determine the possible biological functions of *PbrVHA* genes. Expression patterns of *VHA* genes varied across six tissues, which may be consistent with their diverse physiological roles. *VHA-c* genes in *Arabidopsis* are differentially expressed in expanding cells to support growth. For instance, *AtVHA-c1* and *AtVHA-c2* are highly expressed in roots and shoots, whereas *AtVHA-c3* is mainly expressed in root caps and pollen [42]. *PbrVHA-c1*, *PbrVHA-c3*, and *PbrVHA-c4* were highly expressed in different tissues (Figure 5A), but their expression varied at different pollen tube developmental stages (Figure 6A), indicating the crucial roles of V-ATPases during cell expansion.

The previous studies suggested that *VHA* genes play critical roles in abiotic stress tolerance as well as plant growth and senescence [22,39,43]. The conserved VHA-c subunit plays an important role in plant growth [43]. Transcript analysis of *VHA* genes revealed their possible involvement in citrate accumulation in fruit [18,44]. The functional roles of *PbrVHA* genes at different developmental stages were also investigated in this study. The expression of some genes (*PbrVHA-B2*, *PbrVHA-G1*, *PbrVHA-E3*, *PbrVHA-E1*, and *PbrVHA-d1*) was significantly upregulated at the S3 stage (Figure 5B), suggesting their pivotal roles in pear fruit growth and ripening. There is some evidence that *VHA* genes perform important functions during senescence. Molecular genetic studies have shown that OsPSL1/VHA-A1 and AtVHA-B1 are implicated in leaf senescence [19,45]. In the present study, transcript levels of seven *VHA* genes were downregulated when the pollen tube stopped growing, which was consistent with changes in V-ATPase activity [46]. Proteomic analysis revealed that V-ATPase protein abundance decreased during fruit storage, suggesting that V-ATPase activity is associated with fruit senescence. To further confirm this speculation, we analyzed the expression patterns of *PbrVHA* genes during the storage of “Housui” pear and found that the expression levels of 10 *PbrVHA* genes were significantly downregulated following storage, indicating their role in pear senescence and fruit quality maintenance.

Vacuolar H^+^-ATPases are multi-subunit protein complexes, which were divided into two structurally distinct subcomplexes (V_1_ and V_0_ subunits) [6,12,41]. The water-soluble V_1_ domain was located in the cytosol, which catalyzes ATP hydrolyzing and binding; the membrane-embedded V_0_ domain contained the proton pore and transported protons [41]. The core of the V_1_ domain consists of A and B subunits, whereas the V_0_ domain is composed of subunit VHA-c, VHA-c”, VHA-a and VHA-e. V _0_ and V _1_ complexes are joined by a central stalk (subunits D, F, and d) and three peripheral stators (subunits C, E, G, H, and a) [41]. The assembly of the plant V-ATPase is dependent on all the 13 subunits. The diversity of functions of the V-ATPase may owe to roles of its various subunits and numerous functional regulation sites and motifs [41]. We also found that the expression patterns of some subunits were uniformly expressed, while some were differently expressed (Figure 5 and Figure 6), which suggested that the complexity of the cells coordinates the expression of certain subunits during development and senescence. Genome-wide identification of the *VHA* genes in five Rosaceae species was an important first step in the system research for *VHA* genes, this study laid a foundation for clarify the particular roles of each subunit.

## 4. Materials and Methods

### 4.1. Identification and Characterization of VHA Genes

Protein sequences of previously identified *VHA* gene family members in *Arabidopsis* were downloaded from the Arabidopsis Information Resource (http://www.Arabidopsis.org/) [6]. These amino acid sequences of *A. thaliana* VHA proteins were used as queries to perform BLASTP searches against the National Center for Biotechnology Information Support Center (NCBI) database and the Rosaceae genome, with an e-value cut-off of 1.0 × 10^−10^. In this study, we investigated five Rosaceae species genomes. Pear and Chinese plum genome sequences were downloaded from the Pear Genome Project (http://peargenome.njau.edu.cn/) [27] and *Prunus mume* Genome Project (http://prunusmumegenome.bjfu.edu.cn) [28] respectively. Genomic sequences of peach, apple, and strawberry were downloaded from Phytozome [29]. To further confirm the validity of the identified *VHA* genes, SMART (http://smart.embl-heidelberg.de/) and Pfam (https://pfam.xfam.org) databases were used to search for the functional domains of potential VHA proteins [47]. Molecular weights and pI values of VHA proteins were predicted using ProtParam (http://web.expasy.org/protparam/) [48].

### 4.2. Phylogenetic Analysis

Multiple sequence alignment was performed using ClustalW program based on the amino acid sequences of *A. thaliana*, pear, peach, apple, Chinese plum, and strawberry. A neighbour-joining phylogenetic tree was constructed with 1000 bootstrap replicates using MEGA 6.0 [49]. The diagram was drawn using online tool (iTOL, https://itol.embl.de/), as previously described [50].

### 4.3. Gene Structure and Conserved Motif Analysis

A schematic diagram of the *VHA* gene structure was constructed using Gene Structure Display Server 2.0 (http://gsds.gao-lab.org/index.php) [51] by aligning the coding sequence with the genomic sequence. Conserved motifs of the VHA proteins were analyzed using MEME Version 5.1.0 (http://meme-suite.org/tools/meme) [52]. The optimized parameters were as follows: zero or one site per sequence; optimum width of each motif, 6–100 residues; maximum number of motifs, 30. The secondary structures of VHA proteins were predicted by the online tool NPS@SOPMA (https://npsa-prabi.ibcp.fr/). The 3D models of tertiary structures were constructed using the Swiss-Model (https://www.swissmodel.expasy.org/) [53]. Multiple alignments of V-ATPase proteins from pear and *Arabidopsis* were performed using DNAman soft.

### 4.4. Chromosomal Distribution and Synteny Analysis

Chromosomal distribution of *VHA* genes was analyzed based on the Rosaceae genome annotation data. MCScanX was used for the detection and evolutionary analysis of gene synteny and collinearity among the five Rosaceae species [34]. The Plant Genome Duplication Database (http://chibba.agtec.uga.edu/duplication/index/downloads) was used to identify segmentally duplicated genes among the five Rosaceae genomes [37]. Chromosomal distribution and syntenic relationships of *VHA* genes were plotted using TBtools and Circos. Ka and Ks values of the duplicated pairs were calculated using TBtools [54].

### 4.5. Gene ontology (GO) Items and Expression Pattern Analysis

Global gene expression profiles in various tissues and at different developmental stages were investigated based on publicly available datasets in the NCBI Sequence Read Archive (SRA; https://www.ncbi.nlm.nih.gov/sra) database. RNA-Seq datasets (SRA number: PRJNA590622) were used to determine tissue-specific expression profiles [30]. Raw reads data were used to quantify the expression levels of *PbrVHA* genes in pear fruit at different developmental (SRA numbers: PRJNA309745) or storage stages (SRA numbers: PRJNA309745 and PRJNA597402) [31,33]. Moreover, *PbrVHA* genes involved in pollen tube growth were identified using RNA-Seq datasets (SRA number: PRJNA299117) [32]. Heatmaps were constructed using TBtools [54]. GO annotation analysis of the *PbrVHA* genes was conducted using the Blast2GO program [55].

## 5. Conclusions

In the present study, genome-wide identification and bioinformatic analyses of *VHA* genes in pear and four other Rosaceae species were performed. A total of 159 members of the *VHA* gene family were identified and further classified into 13 subfamilies based on a phylogenetic tree. Most *PbrVHA* genes were derived through WGD under purifying selection. In addition, the expression patterns of *PbrVHA* genes in various tissues and at different fruit developmental or storage stages were investigated using RNA-Seq data. In comparative transcriptomic analyses, some *PbrVHA* genes were differentially expressed during fruit growth and storage, suggesting that *VHA* genes play specific roles in development and senescence. Expression profiles of *PbrVHA* genes were tissue- and cultivar-specific. The present study can serve as a foundation for further investigation of biological functions of *VHA* genes in Rosaceae species.

## Figures and Tables

**Table 1:** Numbers of *VHA* genes in *Arabidopsis thaliana* and five Rosaceae species.

Species	Gene Name Prefix	V_1_ Subunit								V_0_ Subunit					Total
		A	B	C	D	E	F	G	H	a	c	c”	d	e	
*Arabidopsis thaliana*	*At*	1	3	1	1	3	1	3	1	3	5	2	2	2	28
*Fragaria vesca*	*Fve*	1	1	1	1	2	1	2	1	3	3	1	1	2	20
*Prunus persica*	*Ppe*	1	1	1	1	2	2	3	1	3	4	1	1	2	23
*Prunus mume*	*Pmu*	1	1	1	2	2	2	3	1	4	4	1	1	2	25
*Pyrus bretschneideri*	*Pbr*	2	2	1	2	4	1	8	1	7	8	1	2	4	43
*Malus x domestica*	*Md*	2	2	4	2	4	3	6	2	7	9	1	2	4	48

Numbers of VHA genes in the genomes of Arabidopsis thaliana, Fragaria vesca, Prunus persica, Prunus mume, Pyrus bretschneideri, and Malus x domestica are indicated.

**Table 2:** Ka, Ks, and Ka/Ks values of paralogous *PbrVHA* gene pairs.

Gene 1	Gene 2	Ka	Ks	Ka/Ks	Gene Duplication
*PbrVHA-a5*	*PbrVHA-a4*	0.0386	0.1449	0.2664	WGD
*PbrVHA-a7*	*PbrVHA-a5*	0.0389	0.1461	0.2661	WGD
*PbrVHA-a7*	*PbrVHA-a1*	0.0005	0.0113	0.0479	WGD
*PbrVHA-B2*	*PbrVHA-B1*	0.0115	0.1826	0.0634	WGD
*PbrVHA-c5*	*PbrVHA-c3*	0.0054	1.1866	0.0046	WGD
*PbrVHA-c5*	*PbrVHA-c1*	0.0027	0.2052	0.0133	WGD
*PbrVHA-D1*	*PbrVHA-D2*	0.0083	0.2743	0.0306	WGD
*PbrVHA-E1*	*PbrVHA-E3*	0.0481	0.2010	0.2394	WGD
*PbrVHA-G6*	*PbrVHA-G7*	0.0275	0.1560	0.1764	WGD
*PbrVHA-G3*	*PbrVHA-G7*	0.0275	0.1560	0.1764	WGD

Ka: nonsynonymous substitution rate; Ks: synonymous (Ks) substitution rate; Ka/Ks: the non-synonymous/synonymous substitution ratio; WGD whole-genome duplication.

## Supplementary Materials

The following are available online at https://www.mdpi.com/2223-7747/9/12/1661/s1, Figure S1: Logos of PbrVHA protein conserved motifs. A total of 30 motifs were identified by the MEME tool. Figure S2. Secondary structure and multiple alignments of domain analysis of the V-ATPase proteins. A: The secondary structures of VHA proteins were predicted using the online tool NPS@SOPMA (https://npsa-prabi.ibcp.fr/); B: Multiple alignments of the P-loop motif in VHA-A; C: The cross membrane domain and proton binding sites of VHA-c and VHA-c”; D: The cross membrane domain and amino acids identified as essential for H^+^-pumping of VHA-a; E: The cross membrane domain of VHA-e. Alpha helix was colored in red, extend strand was colored in blue, and random coli in purple. The cross membrane domain was marked with a blue box. The cross-membrane domain was analyzed using online tool TMHMM (http://www.cbs.dtu.dk/services/TMHMM/). The P-loop is highlighted with a green box. Amino acids identified as essential for H^+^ -pumping are marked with purple box. Figure S3. The three-dimensional (3D) structural models of V-ATPase proteins in pear. The structure of PbrVHA proteins was constructed using SWISS MODEL. Figure S4: Chromosome location and synteny analysis of the *VHA* genes in the other four Rosaceae species. Gene pairs with a syntenic relationship were joined by a line. A: apple; B: peach; C: Chinese plum; D: strawberry. Figure S5. Chromosomal distribution and orthologous *VHA* gene pairs of the *VHA* genes between pear and strawberry, apple, Chinese plum, peach, respectively. A: Between pear and strawberry; B: Between pear and apple; C: Between pear and Chinese plum; D: Between pear and peach. Figure S6. Expression patterns of *VHA* genes at six key fruit developmental stages of four pear cultivars. Figure S7. Expression profiles of *PbrVHA* genes of five pear cultivars during fruit senescence after harvesting. Table S1: Detailed information of all *VHA* gene family genes identified in the five Rosaceae species
