# Supplementary material for: Genome-Wide Identification of the Vacuolar H+-ATPase Gene Family in Five Rosaceae Species and Expression Analysis in Pear (Pyrus bretschneideri)
Source: Plants (Basel). 2020 Nov 27;9(12):1661. doi: 10.3390/plants9121661 (PMC7761284; doi:10.3390/plants9121661)
Supplement: Supplementary file 1 [file plants-09-01661-s001.zip › Figure S1.docx]

Motif 1:
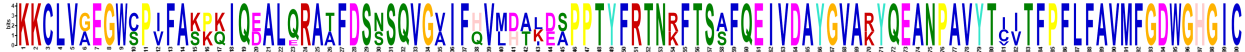


Motif 2:
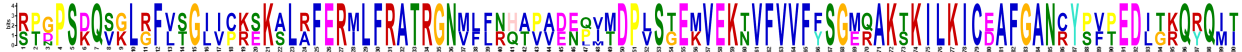


Motif 3:
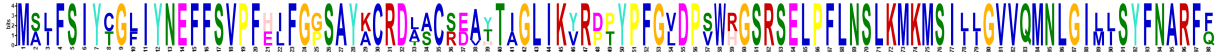


Motif 4:
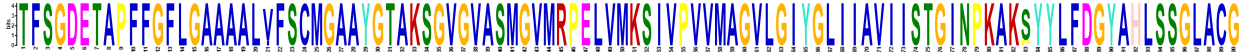


Motif 5:
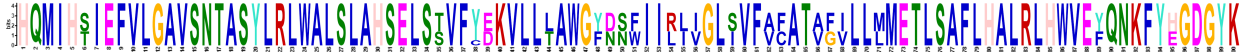


Motif 6:
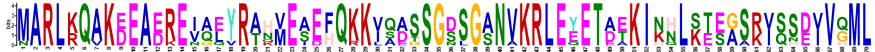


Motif 7:
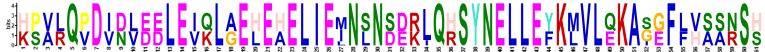


Motif 8:
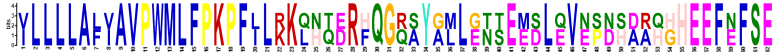


Motif 9:
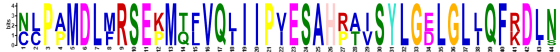


Motif 10:
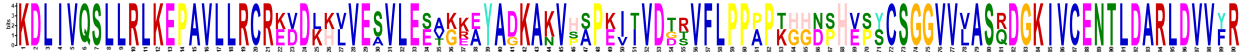


Motif 11:
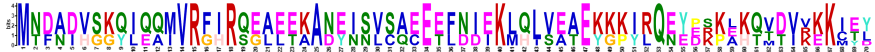


Motif 12:
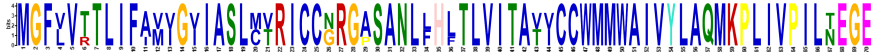


Motif 13:
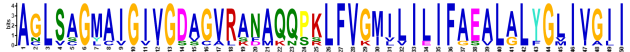


Motif 14:
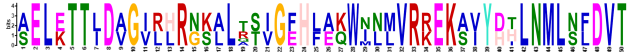


Motif 15:
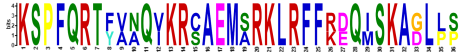


Motif 16:
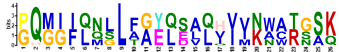


Motif 17:
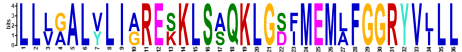


Motif 18:
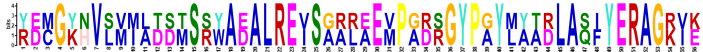


Motif 19:
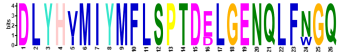


Motif 20:
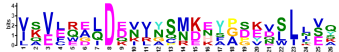


Motif 21:
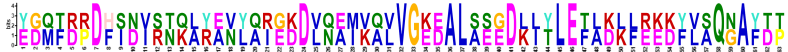


Motif 22:

Motif 23:
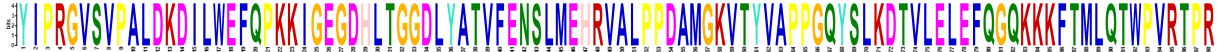


Motif 24:
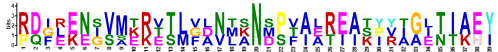


Motif 25:
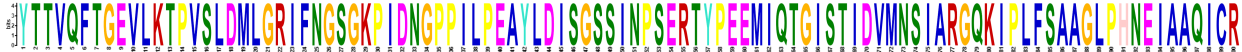


Motif 26:
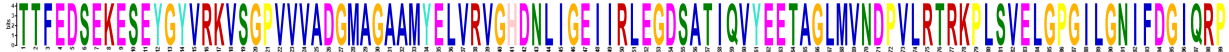


Motif 27:
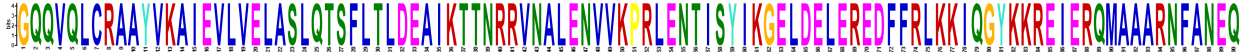


Motif 28:
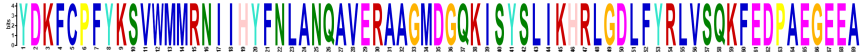


Motif 29:
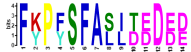


Motif 30:
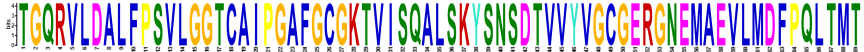


**Figure S1.** Logos of PbrVHA protein conserved motifs. A total of 30 motifs were identified by the MEME tool.
