# Supplementary material for: Genome-Wide Identification of the Vacuolar H+-ATPase Gene Family in Five Rosaceae Species and Expression Analysis in Pear (Pyrus bretschneideri)
Source: Plants (Basel). 2020 Nov 27;9(12):1661. doi: 10.3390/plants9121661 (PMC7761284; doi:10.3390/plants9121661)
Supplement: Supplementary file 1 [file plants-09-01661-s001.zip › Figure S2.docx]

**A**

PbrVHA-A


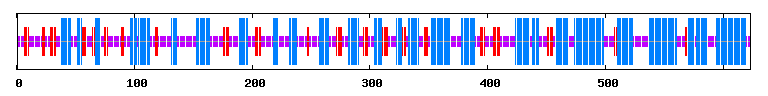


PbrVHA-B1


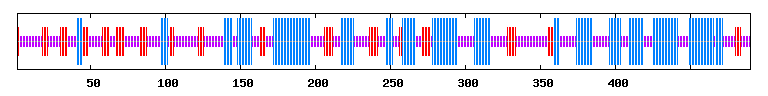


PbrVHA-B2


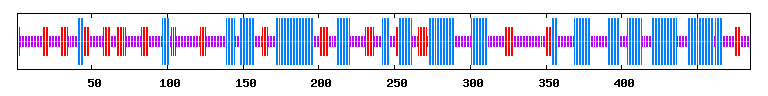


PbrVHA-C


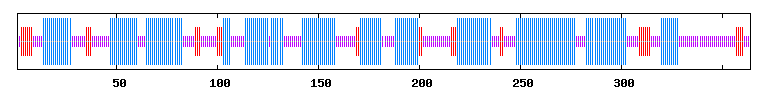


PbrVHA-D1


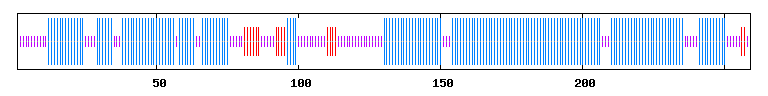


PbrVHA-D2


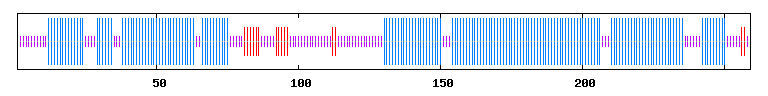


PbrVHA-E1


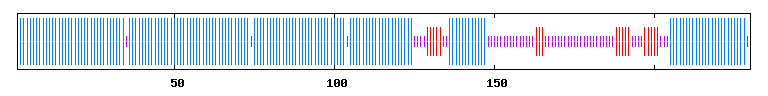


PbrVHA-E2


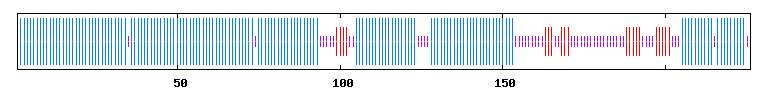


PbrVHA-E3


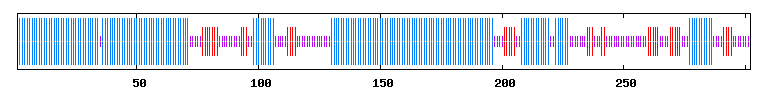


PbrVHA-E4


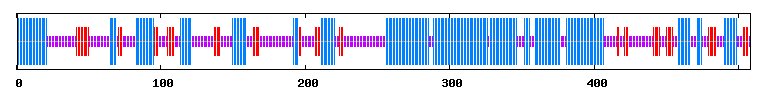


PbrVHA-F


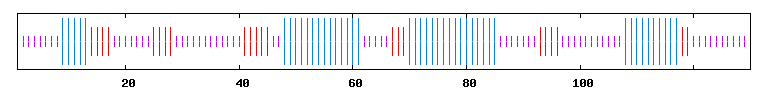


PbrVHA-G1


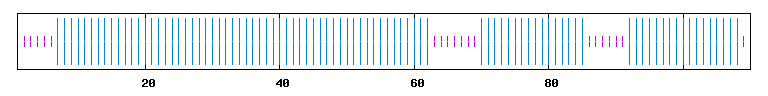


PbrVHA-G2


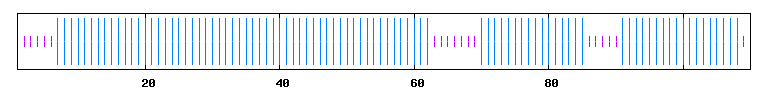


PbrVHA-G3


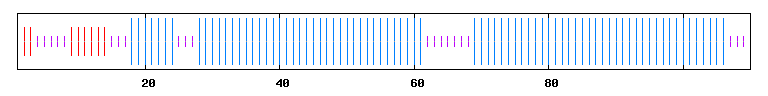


PbrVHA-G4


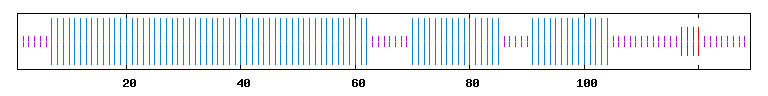


PbrVHA-G5


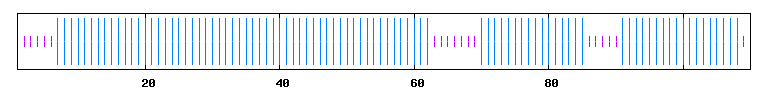


PbrVHA-G6


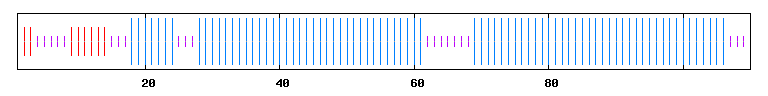


PbrVHA-G7


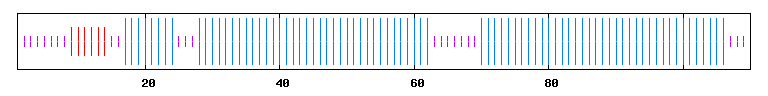


PbrVHA-G8


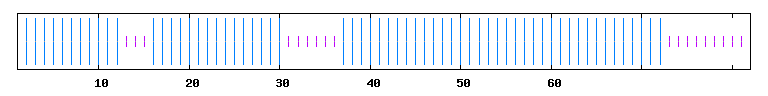


PbrVHA-H


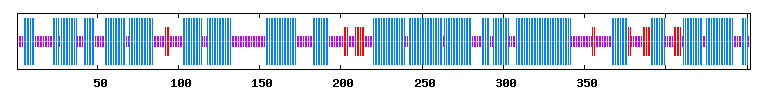


PbrVHA-a1


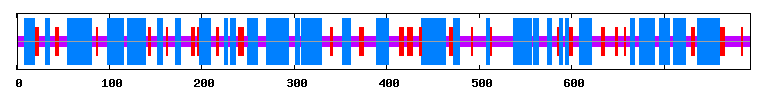


PbrVHA-a2


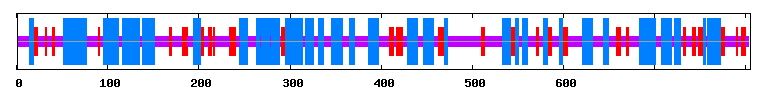


PbrVHA-a3


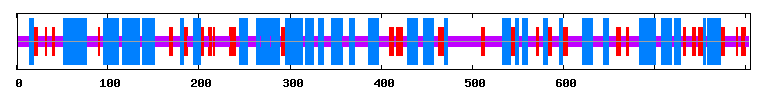


PbrVHA-a4


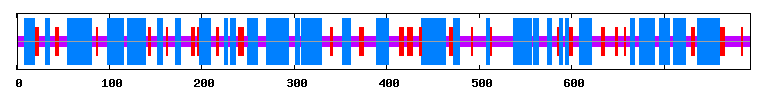


PbrVHA-a5


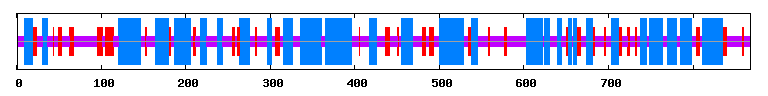


PbrVHA-a6


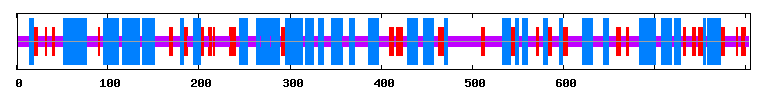


PbrVHA-a7


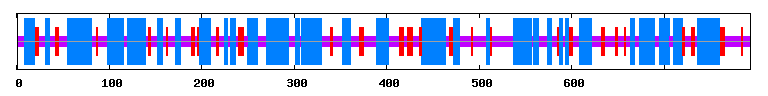


PbrVHA-c1


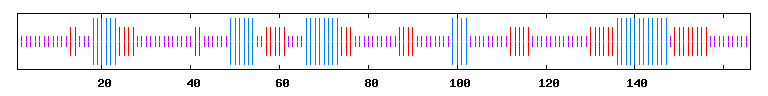


PbrVHA-c2


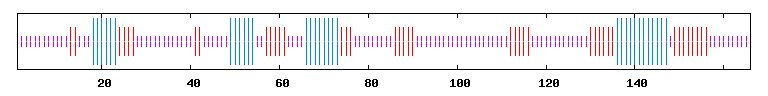


PbrVHA- c3


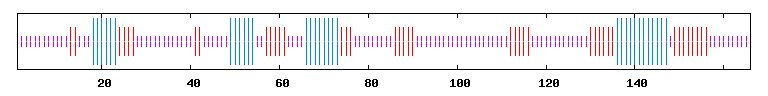


PbrVHA-c4


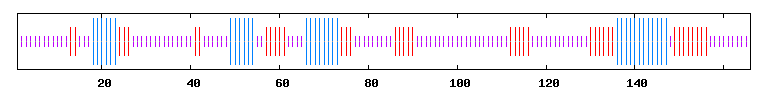


PbrVHA-c5


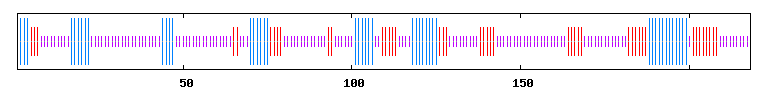


PbrVHA-c6


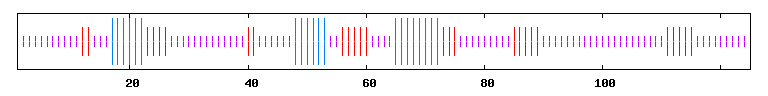


PbrVHA-c7


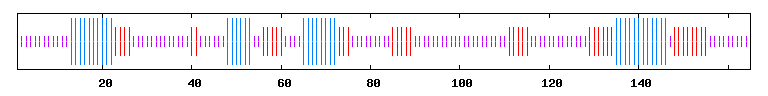


PbrVHA-c8


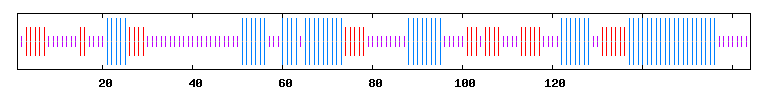


PbrVHA-c”


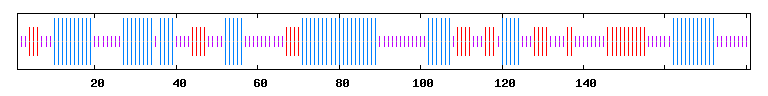


PbrVHA-d1


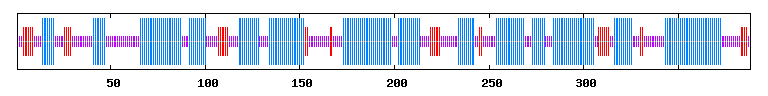


PbrVHA-d2


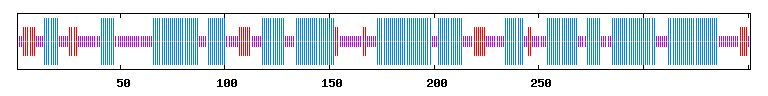


PbrVHA-e1


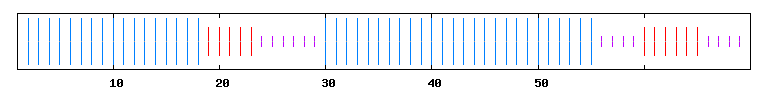


PbrVHA-e2


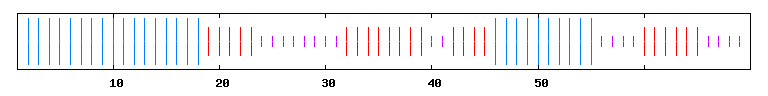


PbrVHA-e3


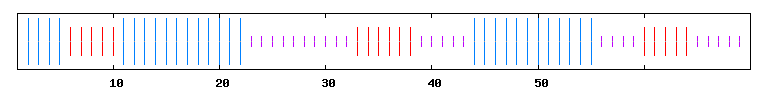


PbrVHA-e4


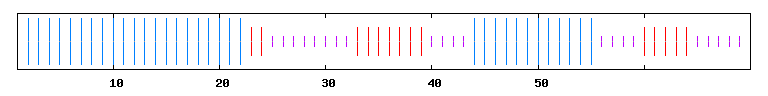


**B**

**C**

**D**

**E**

**Figure S2．** Secondary structure and multiple alignments of domain analysis of the V-ATPase proteins. A: The secondary structures of VHA proteins were predicted using the online tool NPS@SOPMA (<https://npsa-prabi.ibcp.fr/>); B: Multiple alignments of the P-loop motif in VHA-A; C: The cross membrane domain and proton binding sites of VHA-c and VHA-c”; D: The cross membrane domain and amino acids identified as essential for H ^+^-pumping of VHA-a; E: The cross membrane domain of VHA-e. Alpha helix was colored in red, Extend strand was colored in blue and random coli in purple. The cross membrane domain was marked with a blue box.The cross membrane domain was analyzed using online tool TMHMM (http://www.cbs.dtu.dk/services/TMHMM/). The P-loop are highlighted with a green box. Amino acids identified as essential for H ^+^ -pumping are marked with [purple](javascript:;) box.
