# Supplementary material for: Genome-Wide Identification of the Vacuolar H+-ATPase Gene Family in Five Rosaceae Species and Expression Analysis in Pear (Pyrus bretschneideri)
Source: Plants (Basel). 2020 Nov 27;9(12):1661. doi: 10.3390/plants9121661 (PMC7761284; doi:10.3390/plants9121661)
Supplement: Supplementary file 1 [file plants-09-01661-s001.zip › Figure S3.docx]

PbrVHA-A PbrVHA-B1 PbrVHA-B2 PbrVHA-C PbrVHA-D1 PbrVHA-D2


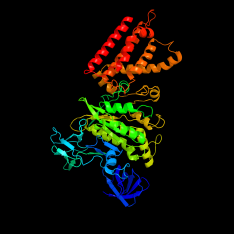

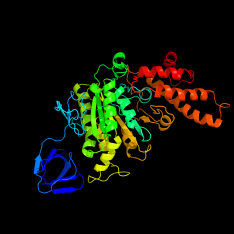

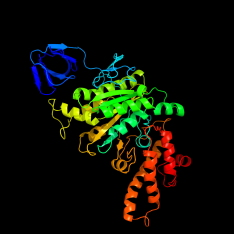

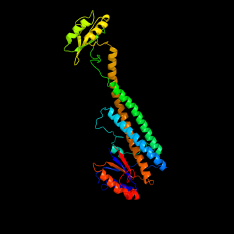

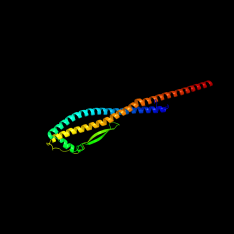

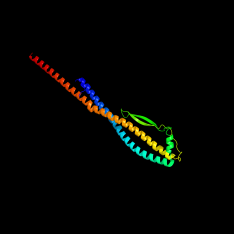


PbrVHA-E1 PbrVHA-E2 PbrVHA-E3 PbrVHA-E4 PbrVHA-F PbrVHA-G1


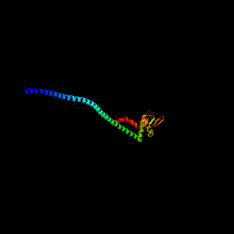

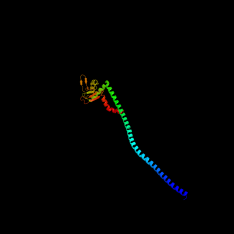

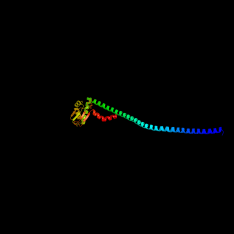

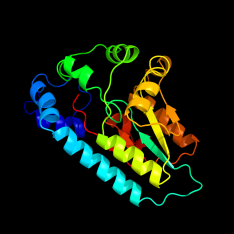

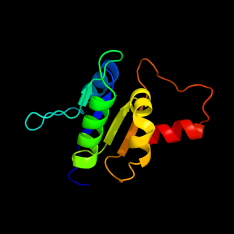

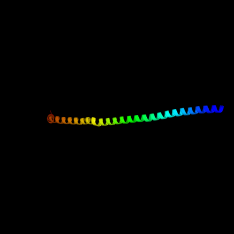


PbrVHA-G2 PbrVHA-G3 PbrVHA-G4 PbrVHA-G5 PbrVHA-G6 PbrVHA-G7


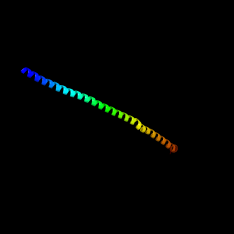

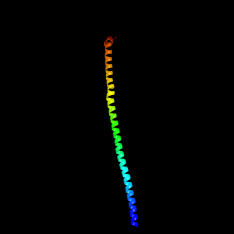

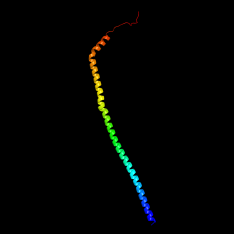

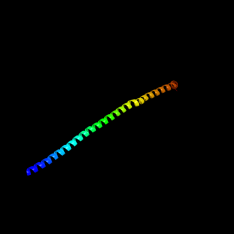

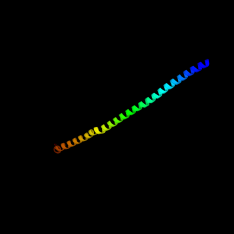

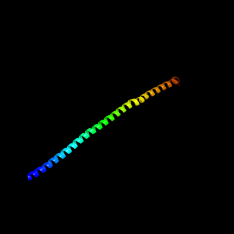


PbrVHA-G8 PbrVHA-H PbrVHA-a1 PbrVHA-a2 PbrVHA-a3 PbrVHA-a4


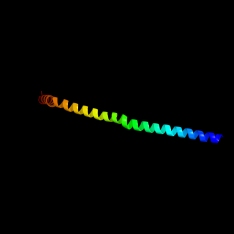

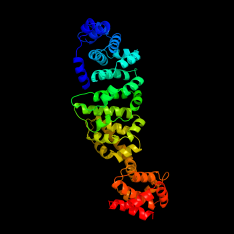

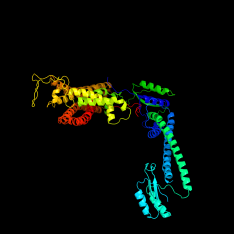

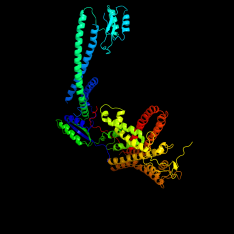

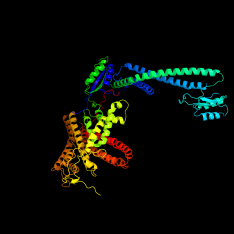

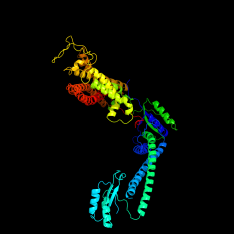


PbrVHA-a5 PbrVHA-a6 PbrVHA-a7 PbrVHA-c1 PbrVHA-c2 PbrVHA- c3


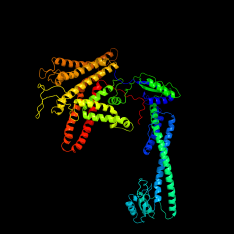

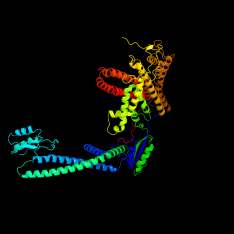

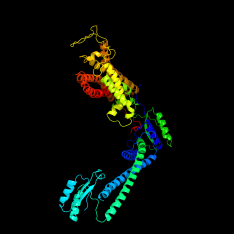

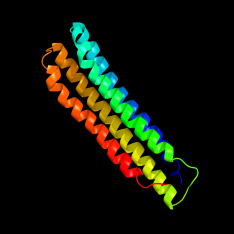

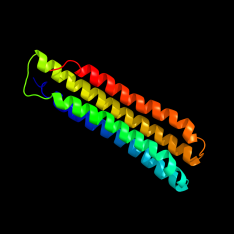

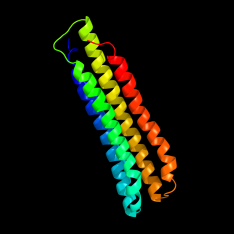


PbrVHA-c4 PbrVHA-c5 PbrVHA-c6 PbrVHA-c7 PbrVHA-c8 PbrVHA-c”


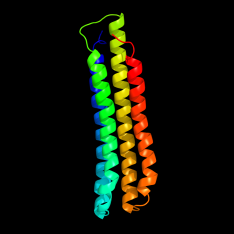

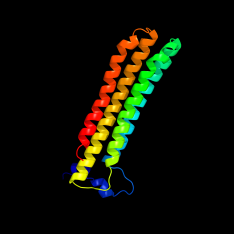

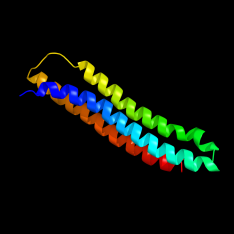

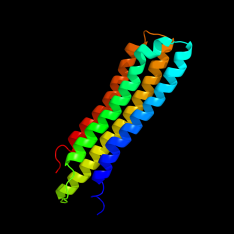

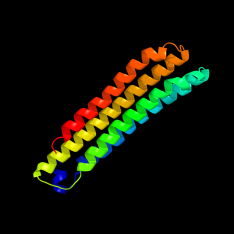

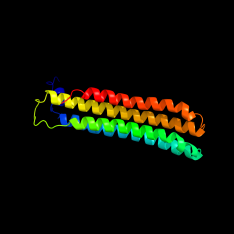


PbrVHA-d1 PbrVHA-d2 PbrVHA-e1 PbrVHA-e2 PbrVHA-e3 PbrVHA-e4


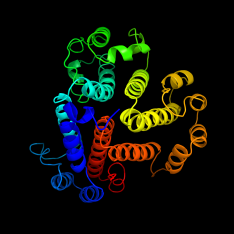

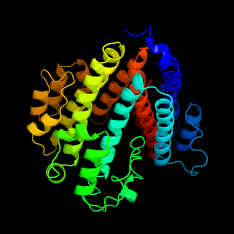

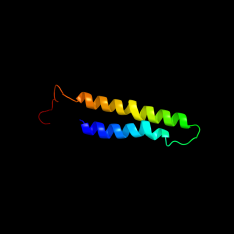

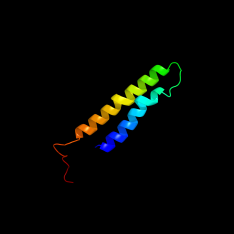

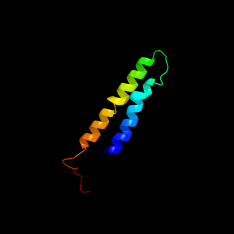

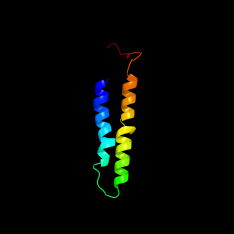


**Figure S3.** The three-dimensional (3D) structural models of V-ATPase proteins in pear. The structure of PbrVHA proteins was constructed using SWISS MODEL.
