# Supplementary material for: Genome-Wide Identification of the Vacuolar H+-ATPase Gene Family in Five Rosaceae Species and Expression Analysis in Pear (Pyrus bretschneideri)
Source: Plants (Basel). 2020 Nov 27;9(12):1661. doi: 10.3390/plants9121661 (PMC7761284; doi:10.3390/plants9121661)
Supplement: Supplementary file 1 [file plants-09-01661-s001.zip › Figure S4.docx]

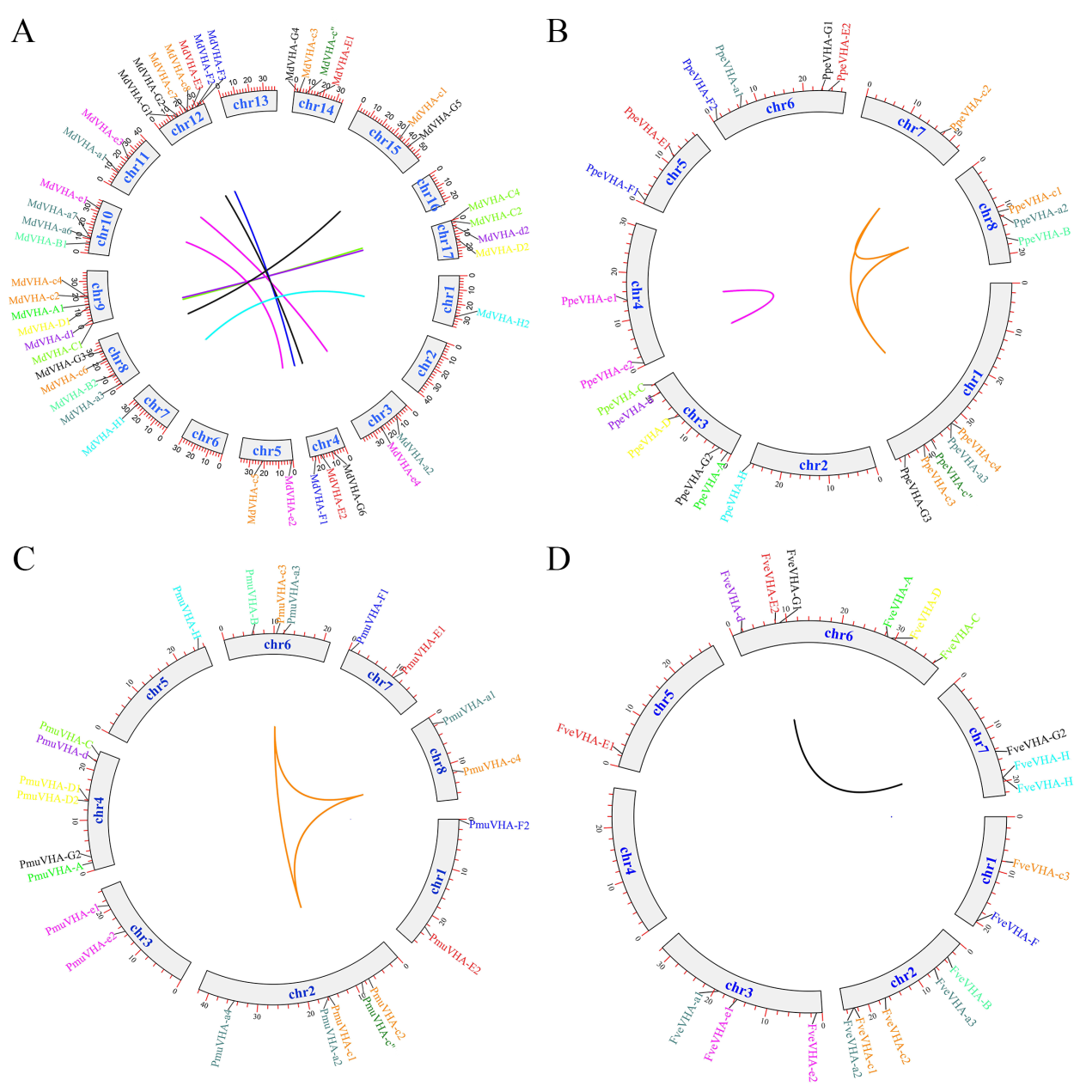


**Figure S4:** Chromosome location and synteny analysis of the VHA genes in the other four Rosaceae species. Gene pairs with a syntenic relationship were joined by a line. A: apple; B: peach; C: Chinese plum; D: strawberry.
