# Supplementary material for: Genome-Wide Identification of the Vacuolar H+-ATPase Gene Family in Five Rosaceae Species and Expression Analysis in Pear (Pyrus bretschneideri)
Source: Plants (Basel). 2020 Nov 27;9(12):1661. doi: 10.3390/plants9121661 (PMC7761284; doi:10.3390/plants9121661)
Supplement: Supplementary file 1 [file plants-09-01661-s001.zip › Figure S5.docx]

**Figure S5.** Chromosomal distribution and orthologous *VHA* gene pairs of the *VHA* genes between pear and strawberry, apple, Chinese plum, peach, respectively. A: Between pear and strawberry; B: Between pear and apple; C: Between pear and Chinese plum; D: Between pear and peach.
