# Supplementary material for: Genome-Wide Identification of the Vacuolar H+-ATPase Gene Family in Five Rosaceae Species and Expression Analysis in Pear (Pyrus bretschneideri)
Source: Plants (Basel). 2020 Nov 27;9(12):1661. doi: 10.3390/plants9121661 (PMC7761284; doi:10.3390/plants9121661)
Supplement: Supplementary file 1 [file plants-09-01661-s001.zip › Figure S6.docx]

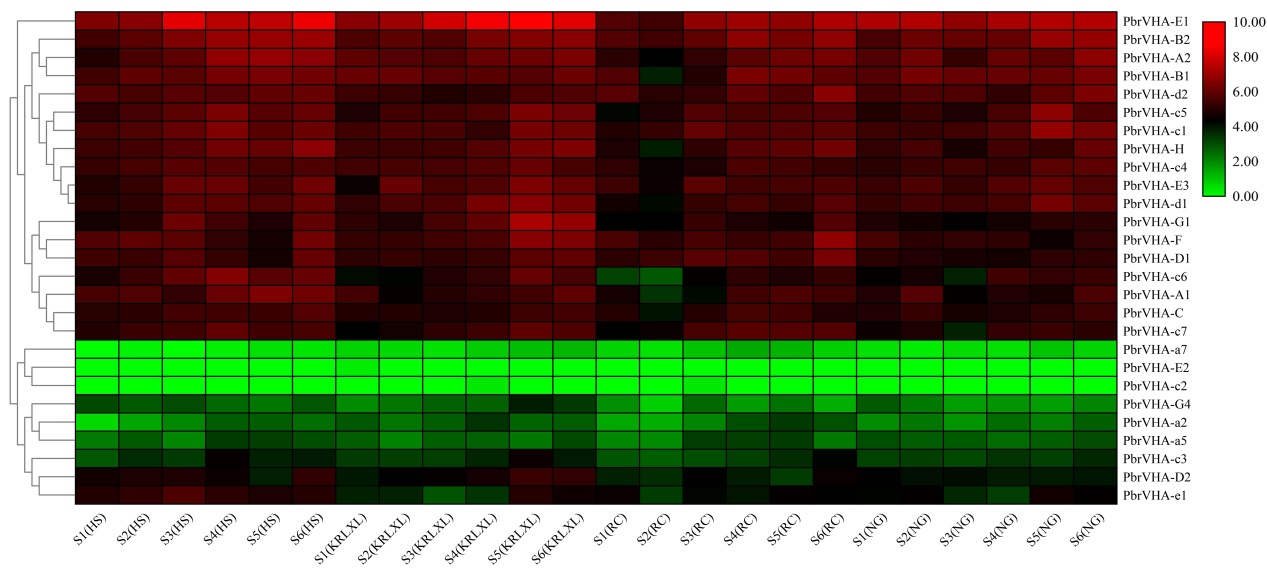


**Figure S6.** Expression patterns of *VHA* genes at six key fruit developmental stages of four pear cultivars. The RNA-seq data of four pear cultivars, ‘Hosui’ (*P. pyrifolia*), ‘Kuerlexiangli’ (*P. sinkiangensis*), ‘Starkrimson’ (*P. communis*), and ‘Nanguoli’ (*P. ussuriensis*) were adapted from SRA database.
